# An Ant Colony Optimization Based Feature Selection for Web Page Classification

**DOI:** 10.1155/2014/649260

**Published:** 2014-07-17

**Authors:** Esra Saraç, Selma Ayşe Özel

**Affiliations:** Department of Computer Engineering, Çukurova University, Balcali, Sarıçam, 01330 Adana, Turkey

## Abstract

The increased popularity of the web has caused the inclusion of huge amount of information to the web, and as a result of this explosive information growth, automated web page classification systems are needed to improve search engines' performance. Web pages have a large number of features such as HTML/XML tags, URLs, hyperlinks, and text contents that should be considered during an automated classification process. The aim of this study is to reduce the number of features to be used to improve runtime and accuracy of the classification of web pages. In this study, we used an ant colony optimization (ACO) algorithm to select the best features, and then we applied the well-known C4.5, naive Bayes, and* k *nearest neighbor classifiers to assign class labels to web pages. We used the WebKB and Conference datasets in our experiments, and we showed that using the ACO for feature selection improves both accuracy and runtime performance of classification. We also showed that the proposed ACO based algorithm can select better features with respect to the well-known information gain and chi square feature selection methods.

## 1. Introduction 

The aim of text classification is to categorize documents into a certain number of predefined classes by using document features. Text classification plays a crucial role in many retrieval and management tasks such as information retrieval, information extraction, document filtering, and building hierarchical directories [1]. When text classification focuses on web pages it is named web classification or web page classification. However, web pages are different from text, and they contain a lot of additional information, such as URLs, links, HTML tags, which are not supported by text documents. Because of this property of web pages, web classification is different from traditional text classification [1].

On the web, classification is used for topic-specific web link selection, analysis of the topical structure of the web, development of web directories, and focused crawling [1]. Previously, people manually constructed some web directories such as Yahoo! [2] and the Open Directory Project [3] and manually assigned class labels to web documents. However, manual classification is time consuming and needs a lot of human effort, which makes it unscalable with respect to the high growing speed of the web. Therefore, there has been great need for automated web page classification systems [4].

A major problem of the web page classification is the high dimensionality of the feature space. We need to select “good” subsets of features from the original feature space to reduce the dimensionality and to improve the efficiency and run time performance of the classification process [5]. Several approaches such as document frequency, information gain, mutual information, chi square analysis, and term strength have been applied to select proper features for text categorization. According to Yang and Pedersen [6] chi square analysis and information gain are more effective for optimizing classification results, and document frequency is less effective but it is scalable and affordable. In Aghdam et al. [7] nature inspired search and optimization algorithms have been applied for feature selection of text classification problem. According to [7], ant colony optimization and genetic algorithms can choose better features than the information gain and chi square analysis, and performance of ant colony optimization is better than the genetic algorithm. For this reason, in this study we applied an ant colony optimization, which was originally developed to solve optimization problems, to select the best features from the web pages for accurate and time efficient classification.

Ant colony optimization (ACO) was inspired from the behavior of real ant colonies, and it is used to solve discrete optimization problems. The first ACO system was introduced by Marco Dorigo in his Ph.D. thesis [8] and was called the ant system (AS). The AS is the result of a research on computational intelligence approaches to combinatorial optimization [8]. The AS was initially applied to the travelling salesman problem [9] and then to other hard problems. The original AS is motivated by the natural phenomenon that ants deposit pheromone on the ground in order to mark some favorable path that should be followed by other members of the colony. The aim of the colony is to find the shortest path between a food source and the nest. The behavior of an ant colony is a good example of a self-organized system such that it is based on positive feedback (i.e., the deposit of pheromone) and negative feedback (i.e., the evaporation of pheromone). Theoretically, if the quantity of pheromone remains the same over time on all edges, no route is chosen. However, because of feedback, a slight variation on an edge allows the edge to be chosen. The algorithm moves from an unstable state in which no edge is stronger than another, to a stable state where the route is composed of the strongest edges. Ant colony optimization has a wide application domain; for example, Liu et al. [10] have used ACO for continuous domains.

In this study, a new ACO based feature selection algorithm, which selects best features from web pages, has been proposed. The contributions of this study are summarized as follows.

- In the earlier studies, only the bag of terms approach is used for feature extraction and ACO is applied to select features among the small number of terms. However, in our study each term in the URLs and in the HTML tags is taken as a different feature. Therefore, a feature is represented as <URL><term>, or <tag><term> pair which we call “tagged terms” representation that yields thousands of features to be extracted from web pages. According to our research in the literature, there exists no ACO based study which works on such large scale feature space.
- In earlier studies, each ant selects features one by one; however, in our study each ant selects a set of features at a time since our feature space is too high, and selecting features one by one increases running time of ACO sharply.
- In earlier studies, only the effect of using features from bag of terms approach has been studied. In this study, the effect of using features only from URLs, from <title> tags, and tagged terms, as well as bag of terms approach, is investigated. We also study the effects of HTML tags on classification performance.

This paper is organized as follows. In Section 2, we give related work on ACO based feature selection and web page classification.  Section 3 describes our ACO-based feature selection algorithm.  Section 4 includes the datasets used, the experiments performed, their results, and discussions.  Section 5 concludes the study and gives some future work.

## 2. Related Work

In this section, we give a brief review of web page classification, feature selection, and ant colony optimization for feature selection.

### 2.1. Web Page Classification

Classification is the process of assigning predefined class labels to some unseen or test data. For this purpose, a set of labeled data is used to train a classifier which is then used for labeling unseen data. This classification process is also defined as a supervised learning [11]. The process is not different in web page classification such that there is one or more predefined class labels and a classification model assigns class labels to web pages which are in fact hypertext and have many features such as textual tokens, markup tags, URLs, and host names in URLs that are meaningful for classifiers. As web pages have additional properties, their classification has several differences from traditional text classification [1].

Web page classification has some subfields like subject classification and functional classification [1]. In subject classification, classifier is concerned with the content of a web page and tries to determine the “subject” of the web page. For example, categories of online newspapers like finance, sport, and technology are instances of subject classification. Functional classification on the other hand deals with the function or type of the web page. For example, determining whether a web page is a “personal homepage” or a “course page” is an instance of a functional classification. Subject and functional classification are the most popular classification types [1].

Classification can be divided into binary classification and multiclass classification according to the number of classes [1]. In binary classification, there is only one class label. Classifier looks for an instance and assigns it to the specific class or not. Instances of the specific class are called relevant, and the others are named nonrelevant. If there is more than one class, this type of classification is called multiclass classification [1]. The classifier also assigns an instance to one of the multiple classes. In our study, we focus on functional classification of web pages, and we make binary classification since it is the basis of the focused crawlers [12, 13] or topical crawlers [14] of the search engines. The techniques developed in this study can also be used for subject classification and/or multiclass classification of web pages.

### 2.2. Feature Selection for Web Page/Text Classification

Feature selection is the one of the most important steps in classification systems. Web pages are generally in HTML format. This means that web pages are semistructured data, as they contain HTML tags and hyperlinks in addition to pure text. Because of this property of web pages, feature selection in web page classification is different than traditional text classification. Feature selection is generally used to reduce dimension of data with tens or hundreds of thousands of features which would be impossible to process further. A major problem of web page classification is the high dimensionality of the feature space. The best feature subset contains the least number of features that most contribute to classification accuracy and efficiency.

To improve the performance of web page classification, several approaches that are imported from feature selection for text classification have been applied. Information gain [11], mutual information [15], document frequency [6], and term strength [16] are the most popular traditional feature selection techniques. Information gain (IG) measures the amount of information in bits about the class prediction, if the only information available is the presence of a feature and the corresponding class distribution. Concretely, it measures the expected reduction in entropy [11].

Mutual information (MI) was first introduced by Shannon [15] in the context of digital communications between discrete random variables and was generalized to continuous random variables. Mutual information is considered as an acceptable measure of relevance between two random variables [17]. Mutual information is a probabilistic method which measures how much information the presence/absence of a term contributes to making the correct classification decision on a class [18].

Document frequency (DF) is the number of documents in which a term occurs in a dataset. It is the simplest criterion for term selection and easily scales to a large dataset with linear computational complexity. It is a simple but effective feature selection method for text categorization [6].

Term strength (TS) has been proposed and evaluated by Wilbur and Sirotkin [16] for vocabulary reduction in text retrieval. Term strength is also used in text categorization [19, 20], such that it predicts term importance based on how commonly a term is likely to appear in “closely-related” documents. TS uses training set of documents to derive document pairs whose measured similarity according to the cosine value of the two document vectors is above a threshold. Then, “term strength” is computed based on the predicted conditional probability that a term occurs in the second half of a pair of related documents given that it occurs in the first half. The above methods namely the IG, the DF, the MI, and the TS have been compared by Yang and Pedersen [6] by using the kNN classifier on the Reuters [21] corpus. According to [6], IG is the most effective method with 98% feature reduction; DF is the simplest method with the lowest cost in computation and it can be credibly used instead of IG if computation of this measure is too expensive.

In addition to traditional feature selection methods, swarm intelligence techniques are also popular to be used for feature selection. In this study, we review the application of ACO for feature selection in general classification problems and web/text classification domains.

### 2.3. Ant Colony Optimization for General Classification Problems

Jensen and Shen [22] have proposed a hybrid approach which is a combination of fuzzy rough set and ACO to select features from the Water Treatment Plant database. Fuzzy rough set dependency measure is used for features which are more informative in the currently given selected subset. The number of features is reduced from 38 to 10 with fuzzy rough sets, and then it is further reduced from 10 to 9.5 with ACO. Dependency measure is used as the stopping criteria, and C4.5 is employed for classification. Instead of accuracy and* F*-measure values only the training and testing errors are presented. The error rates in training with no feature reduction, fuzzy rough set reduction, and ant fuzzy rough set reduction are 1.5%, 10.8%, and 6.5%, respectively. They have observed 19.1%, 25.2%, and 22.1% testing errors with no feature reduction, fuzzy rough set reduction, and ant fuzzy rough set reduction, respectively.

Chen et al. [23] have proposed a rough set approach for feature selection based on ACO. Mutual information is used as heuristic information. Feature selection is started with a feature core rather than a random feature which causes complete graph to become in a smaller form. Feature reduction is finished after the core has been found. They have studied the UCI dataset with C4.5 classifier and achieved 98.2% average accuracy for classification.

Huang [24] has used classification accuracy and feature weights of the constructed SVM classifier to design the pheromone update in ACO based feature selection. In this study the UCI and simulated datasets are used and 94.65% average accuracy value is obtained.

Sivagaminathan and Ramakrishnan [25] have proposed a hybrid approach which is a combination of the neural networks and ACO for feature selection. Neural networks are used for error prediction and classification. In the experiments 3, 5, 8, 10, and 12 ants are used for the medical diagnosis dataset which contains 21 to 34 features, and 77.50% average accuracy on breast cancer (Wisconsin prognostic) and 98.22% average accuracy on thyroid disease datasets are achieved.

Vieira et al. [26] have proposed two separate ant colonies combined with fuzzy models for feature selection; one colony is used for minimizing number of features, and the other one is employed to minimize classification error. In this study, the first colony determines the number of features and the second one selects the features. A fuzzy model is used as a classifier and 96.4% average accuracy on breast cancer (Wisconsin prognostic) dataset is observed.

Nemati and Basiri [27] have proposed an ACO based method for a speaker verification system. Gaussian mixture model universal background model (GMM-UBM) is used as a classifier over the TIMIT corpora and equal error rate (EER) of the classification is taken as evaluation criteria. 4.56%, 2.634%, and 3.679% EER values are observed with GMM-UBM, ACO, and GA methods, respectively.

Kabir et al. [28] have combined ACO with neural networks where neural networks are used as classifier. The proposed study contains both wrapper and filter methods such that a probabilistic formula for random selection and determination of subset size is used. Eight well-known cancer datasets are used in the experiments and 98.91% average accuracy is achieved.

Akarsu and Karahoca [29] have used ACO for clustering and feature selection. Ant colony clustering technique is used to segment breast cancer dataset. To remove irrelevant or redundant features from the dataset, sequential backward search technique is applied. Feature selection and clustering algorithms are incorporated as a wrapper. The results showed that the accuracy of the FS-ACO clustering approach is better than the filter approaches. They have compared their study with the clustering algorithm in Weka with respect to the sum of squared error value. According to the experimental evaluation, the sum of squared error is 732 for Weka and 758 for the proposed method.

Al-Ani [30] has proposed an ACO based subset search procedure for speech classification problem. Local importance of a given feature is measured by using the mutual information evaluation function, and only the best* k* subsets are used to update the pheromone. First iteration starts with *m* features. The second and following steps start with *m* − *p* features that are selected randomly from the previous* k-*best subsets. *p* is a numeric value which changes from 1 to *m* − 1. They have studied TIMIT corpora with ANN classifier. The average classification accuracy of ACO, GA, and SFS over all the cases is 84.22%, 83.49%, and 83.19%, respectively.

Wang et al. [31] have developed an ACO based feature selection, which employs SVM classifier, to find the best feature subset for the UCI dataset. The experiments are performed with 5, 10, 15, 20, and 25 ants, and the best feature subset is found with 15 ants. In the experimental evaluation 94.83% average accuracy for Wine dataset and 79.57% average accuracy for Image Segment dataset are observed.

Jain and Singh [32] have modified probability function of ANT with exponential function for feature selection. Two modified ant algorithms are applied to a number of problems and the results are compared with those obtained by applying the original ACO and genetic algorithm, and the same results but with better execution time are observed.

Abd-Alsabour and Randall [33] have proposed a wrapper based system for feature selection. In the experiments, UCI dataset and SVM classifier are used. In the proposed feature selection algorithm, the number of selected features is not fixed, so the length of each ant's feature subset may differ. By using the features selected by ants, 1 and 0.8584 classification accuracy values are observed for Wine and Vehicle datasets, respectively.

Rasmy et al. [34] have proposed a hybrid approach for feature selection. They have used ACO for selecting features and ANTMiner for classification. In their ACO, ants start choosing a node (i.e., feature) randomly, after that classifier performance and length of selected feature vectors are adopted as heuristic information for ACO. In this study, UCI dataset having 150 to 560 features is used and the number of features is reduced to 9 and 70 for Diabetes and Analcatdata classes, respectively. After feature selection, 94.4% and 85% average classification accuracy is achieved for Sonar and Diabetes datasets, respectively.

### 2.4. Ant Colony Optimization for Text and Web Classification

AntMiner [35] is the first study that uses the ACO in the web page classification domain. Holden and Freitas [36] have been inspired by AntMiner [35] and used the ant colony paradigm to find a set of rules that classify the web pages into several categories. They have no prior assumptions about which words in the web pages to be classified can be used as potential discriminators. To reduce data rarity, they use stemming which is a technique in which different grammatical forms of a root word are considered as equivalent such that* help, helping, *and* helped *are taken as* help*. Holden and Freitas [36] have also gathered sets of words if they are closely related in the WordNet electronic thesaurus. They have compared their AntMiner with the rule inference algorithms C4.5 and CN2. They have found that AntMiner is comparable in accuracy and forms simpler rules with respect to C4.5 and CN2. The best result of AntMiner is 81.0% classification accuracy and this result is obtained when WordNet generalization is used with Title features.

Aghdam et al. [7] have proposed an ACO based feature selection algorithm for text classification. The features selected by an ant are evaluated according to the classifier performance and the feature subset length. In the experiments, it is assumed that classifier performance is more important than subset length, so that they assign 80% and 20% weights to classifier performance and the subset length, respectively. To measure classification performance, a simple* k* nearest neighbor classifier is used in the experiments. The performance of the proposed algorithm is compared with the performance of a genetic algorithm, information gain, and chi square analysis on the task of feature selection in Reuters-21578 dataset [21]. Their experimental evaluation showed the superiority of the ACO based feature section over genetic algorithms, information gain, and chi square analysis methods. They studied only bag of terms feature extraction method and observed 89.08% microaverage* F*-measure value for Reuters-21578 dataset.

Jensen and Shen [37] have proposed an ACO enhanced fuzzy rough feature selection for web page classification. Terms extracted from web pages are weighted according to the *tf*∗*idf* weighting scheme. In the proposed ACO based feature selection algorithm, each subset selected by each ant is evaluated by a fuzzy-rough measure of the selected subset. The pheromone values are updated according to this measure and the length of the selected subset. After selecting the best feature set, the web pages are then classified. The experiments are performed on a small dataset which contains 280 web pages collected from Arts & Humanities, Entertainment, Computers & Internet, Health, Business & Economy categories of Yahoo directory and it is observed that ACO based feature selection performs the highest degree of reduction in the feature space with minimal loss of information.

Janaki Meena et al. [38] have used ACO for feature selection and naïve Bayes for classification over the 20 Newsgroup dataset. The ratio between observed frequency and expected frequency of the term is applied as a heuristic measure to the features extracted according to the bag of terms method. Map reduce is used for parallelization. Experiments are performed with 500 ants and 150 iterations. For talk.politics.mideast dataset, recall and precision values are 0.94 and 0.68, respectively.

Mangai et al. [39] have studied the WebKB dataset. Features are selected with Ward's minimum variance measure, information gain, and *tf*∗*idf* methods. Ward's minimum variance measure is first used to identify clusters of redundant features in a web page. In each cluster, the best representative features are retained and the others are eliminated. Removing such redundant features helps to minimize the resource utilization during classification. After clustering process, features are selected from these clusters, then kNN, SVM, naïve Bayes, and C4.5 classifiers are used with 10-fold cross validation for classification. Course web pages are used as positive instances and student web pages are used as negative instances. The proposed method of feature selection is compared with other common feature selection methods. Experiments showed that the proposed method performs better than most of the other feature selection methods in terms of reducing the number of features and the classifier training time. 95.00% and 95.65% accuracy values are achieved with kNN and SVM classifiers, respectively.

Our proposed system is different from the above studies in the following respects.

- We use ACO for selecting a predefined number of features from the large feature space extracted from the web pages instead of selecting features one by one. Our feature selection method chooses features as feature groups. Since using a single feature to determine the class of a web page is not enough, also, including features one by one to the selected feature list of each ant increases run time of ACO because all the computations for pheromone update must be repeated for each selection process. However, when we choose a set of features, we perform the necessary computations just once for each selected set.
- We adopt ACO pheromone update formula for web page classification such that our ants are not blind as in the original ACO's ants; we feed them with *tf*∗*idf* value of each term. So, they have an idea about terms before selecting features.
- We have investigated effects of using features from URLs, <title> tags, tagged terms, and bag of terms on the classification performance.
- We have used larger datasets with larger feature spaces.
- We have also investigated which tags are more important for web page classification.

## 3. Ant Colony Optimization for Feature Selection

**Figure 1:**
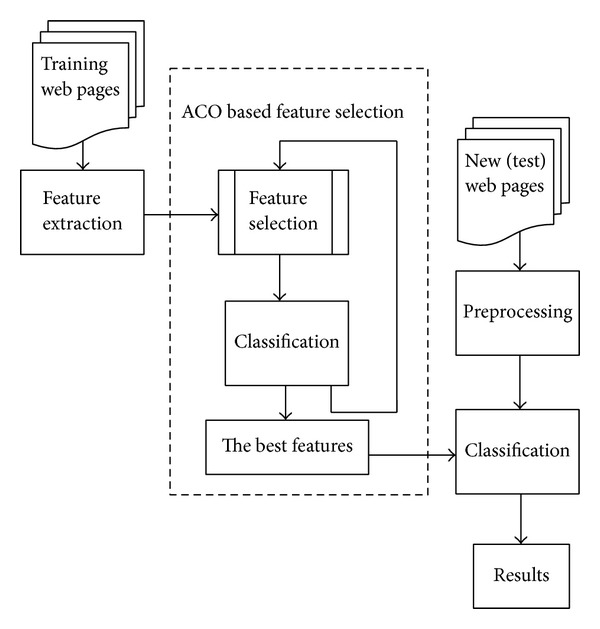
Architecture of the proposed system.

This section includes our ACO based feature selection and classification system. The main structure of the proposed system is shown in Figure 1. Our system consists of feature extraction, ACO based feature selection, and classification components. The training dataset is prepared according to binary class classification problem. From the training dataset, features are extracted, after that the best subset of features is selected by our ACO algorithm and then, by using the selected best features, new web pages are classified with Weka [40] data mining software implemented in Java. The components of our proposed system are explained in detail in the following subsections.

### 3.1. Feature Extraction

In the feature extraction phase all terms from the <title>, <h1>, <h2>, <h3>, <a>, <b>, <i>, <em>, <strong>, <p>, and <li> tags which denote title, header at level 1, header at level 2, header at level 3, anchor, bold, italic, emphasize, strong, paragraph, and list item, and additionally URL addresses of web pages are used. According to the experimental results of the earlier studies [41–43], these tags have useful information and should be used during feature extraction. To extract features, all the terms from each of the above mentioned tags and URL addresses of the relevant pages in the training set are taken. After term extraction, stopwords are removed and the remaining terms are stemmed by using the Porter's stemmer [44]. The stemmed terms and their corresponding tags form our whole feature set. The details of the feature extraction step are presented in Section 4.3.

### 3.2. Feature Selection

**Figure 2:**
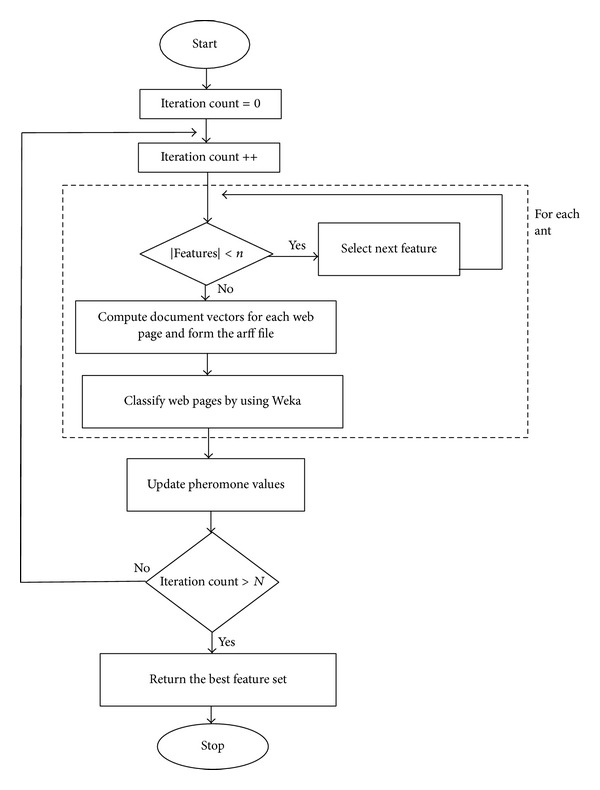
Flow chart of the proposed ACO algorithm.

After the feature extraction step, the (sub)optimum subset of features is selected with our ACO based feature selection algorithm. The flowchart of our proposed ACO algorithm is presented in Figure 2.

In our proposed method, each feature represents a node, and all nodes are independent of each other. Nodes (i.e. features) are selected according to their selection probability *P*_*k*_(*i*) which is given in (1). Initially, all nodes have the same selection probability:

(1) $$\begin{array}{c} P_{k}\left(i\right)=\frac{{\left[\tau \left(i\right)\right]}^{\alpha }{\left[\eta \left(i\right)\right]}^{\beta }}{\sum_{l\in N_{i}^{k}}{\left[\tau \left(l\right)\right]}^{\alpha }{\left[\eta \left(l\right)\right]}^{\beta }}, \end{array}$$

where *η*(*i*) is equal to the document frequency of feature *i* which is the number of documents in the training set that contains feature *i* and represents heuristic information available to the ants. *N*_*i*_^*k*^ is the “feasible” neighborhood of ant *k*, that is, all features as yet unvisited by ant *k*. *τ*(*i*) is the pheromone trail value of feature *i*. Parameters *α* and *β* determine the relative influence of heuristic and pheromone information respectively. The pheromone values, and parameters *α* and *β* are initialized according to [45] which have shown that 1 is the best value for *α* and *β*, and 10 is suitable for initial pheromone trail value. After all the ants have built a complete tour, the pheromone trail is updated according to the* global update rule* which is given in (2) as

(2) $$\begin{array}{c} \tau \left(i\right)=\rho \tau \left(i\right)+\overset{n}{\underset{k=1}{\sum }}\Delta \tau_{k}\left(i\right), \end{array}$$

where *ρ* denotes pheromone evaporation parameter which decays the pheromone trail, and *n* is the number of ants. According to [45], *ρ* value is selected as 0.2. The specific amount of pheromone, Δ*τ*_*k*_(*i*), that each ant* k* deposits on the trail is given by (3) as

(3) Δτk(i)={2BkLkif  node  i  is  used  by  elithist  ant  kBkLkif  node  i  is  used  by  any  ant  k0otherwise.

In (3), *L*_*k*_ is the* F*-measure value of ant* k'*s feature subset, and *B*_*k*_ is the unit pheromone value. This means that the higher the* F*-measure of the ant's selected subset, the more the pheromone deposited on the features used in the subset, and these features are more likely to be selected in the next iteration.

The proposed ACO based feature selection algorithm works as follows. Initially all features have the same selection probability *P*_*k*_(*i*) value. According to *P*_*k*_(*i*) value of each feature *i*, an ant *k* chooses *n* features. A roulette wheel selection algorithm [46] is used to select each of the *n* features.

When an ant *k* chooses *n* features, the web pages in the training dataset are classified with respect to the selected *n* features by using the C4.5 classifier (i.e., the J48 classifier) of Weka data mining tool. To classify the web pages in the training dataset, *tf*∗*idf* values where *tf* is the term frequency and *idf* is the inverse document frequency of the selected features for each web page are taken as the feature values.

The J48 classifier is an open source Java implementation of the C4.5 algorithm in the Weka data mining tool. C4.5 is a well-known decision tree algorithm, and it is an extension of Quinlan's earlier ID3 algorithm [47]. It builds decision trees from a set of training data using an extension of information gain known as gain ratio. After making classification by using the selected features, the classification performance is measured with respect to* F*-measure [48] value which is given in (4) as

(4) $$\begin{array}{c} F\text{-measure}=\frac{2*\text{recall}*\text{precision}}{\text{recall}+\text{precision}}. \end{array}$$

*F*-measure is a combination of precision and recall such that* recall* is the proportion of web pages which are classified as class *C*_*i*_, among all web pages which truly have class *C*_*i*_, and* precision* is the proportion of the web pages which truly have class *C*_*i*_ among all those which are classified as class *C*_*i*_. In earlier studies, researchers measured performance with respect to* F*-measure value. To comply with the standards on this issue,* F*-measure value is chosen as the performance metric in this study. The above feature selection and* F*-measure value computations are repeated for all ants. After these computations, an ant is chosen as an elitist ant which has the highest* F*-measure value. Then, the pheromone values are updated based on (2) and (3). This process is repeated a predetermined number of times (i.e.,* N*). Finally, the feature subset having the best* F*-measure value is chosen as the best feature set which can then be used for classifying new (unseen) web pages.

## 4. Experimental Evaluation and Results

This section includes the datasets, namely, the WebKB and the Conference that were used in this study, the experiments performed, and their results.

### 4.1. WebKB Dataset

The WebKB dataset [49] is a set of web pages collected by the World Wide Knowledge Base (Web->KB) project of the CMU [50] text learning group and has been downloaded from The 4 Universities Dataset Homepage [51]. These pages are collected from computer science departments of various universities in 1997 and manually classified into seven different classes, namely, student, faculty, staff, department, course, project, and others. For each class, the collection contains web pages from four universities which are Cornell, Texas, Washington, Wisconsin universities, and other miscellaneous pages collected from other universities.

The 8,282 web pages are manually classified into the seven categories such that the student category has 1641 pages, faculty has 1124, staff has 137, department has 182, course has 930, project has 504, and other contains 3764 pages. The class* other* is a collection of pages that are not deemed as the “main page” and are not representing an instance of the previous six classes. The WebKB dataset includes 867 web pages from Cornell University, 827 pages from Texas University, 1205 pages from Washington University, 1263 pages from Wisconsin University, and finally 4120 miscellaneous pages from other universities.

From the WebKB dataset* Project, Faculty, Student,* and* Course *classes are used in this study. As* Staff *and* Department *classes have small number of positive examples, they are not considered. Training and test datasets are constructed as described in the WebKB project website [51]. For each class, training set includes relevant pages which belong to randomly chosen three universities and others class of the dataset. The fourth university's pages are used in the test phase. Approximately 75% of the irrelevant pages from others class are included in the training set and the remaining 25% of them are included in the test set. The number of web pages in the train and test part of the WebKB dataset, which is used in this study, is given in Table 1. For example, the Course class includes 846 relevant and 2822 irrelevant pages for the training phase and 86 relevant and 942 irrelevant pages for the test phase.

### 4.2. Conference Dataset

The Conference dataset consists of the computer science related conference homepages that were obtained from the DBLP website [52]. The conference web pages are labeled as positive documents in the dataset. To complete the dataset, the short names of the conferences were queried using the Google search engine [53] manually, and the irrelevant pages in the result set were taken as negative documents. The dataset consists of 824 relevant and 1545 irrelevant pages which are approximately 2 times of the relevant pages.

In the Conference dataset, approximately 75% of both relevant and irrelevant pages are taken as the training set, and the remaining 25% of the relevant and irrelevant pages are included into the test set. The number of web pages in the train and test part of the Conference dataset is given in Table 2.

### 4.3. Feature Extraction

In the feature extraction phase all <title>, <h1>, <h2>, <h3>, <a>, <b>, <i>, <em>, <strong>, <li>, and <p> tags, text content, and URL addresses of web pages are used. All the terms from each of the above mentioned tags and URL addresses of the relevant web pages in the training set are taken. After term extraction, stopword removal and Porter's stemming algorithm [44] are applied. Each stemmed term and its corresponding tag or URL pair forms a feature. For example, a term “program” in a <title> tag, in a <li> tag, or in a URL is taken as a different feature and this feature extraction method is called “tagged terms” method. Terms from similar HTML tags, for example, <strong>, <b>, <em>, and <i>, are grouped together to reduce the feature space.

In this study, features are selected from four different feature sets for each class. In the first set, features are extracted only from the URL addresses of web pages. Secondly, only <title> tags are used for feature extraction. In the third feature extraction method, all terms that appear in the web pages regardless of their HTML tag are used as features. In other words, a term which appears in the document regardless of its position is taken as a feature. This feature extraction approach is called ‘‘bag-of-terms” method. Finally, all terms that appear in each of the above listed HTML tags are used as features. In other words, a term which appears in different HTML tags is taken as a different feature (i.e., tagged terms).

The number of features varies according to the dataset and the feature extraction method used. Numbers of features for each class of all datasets with respect to the feature extraction method used are shown in Table 3. As an example, 33519 features are extracted when tagged terms method are used for the Course class. When only the <title> tag is considered, the number of features extracted reduces to 305 for this class.

### 4.4. Experimental Setup

In this study, Perl script language was used for the feature extraction phase, and our ACO based feature selection algorithm was implemented in Java programming language under Eclipse environment. The proposed method was tested under Microsoft Windows 7 operating system. The hardware used in the experiments has 16 GB of RAM and Intel Xenon E5-2643 3.30 GHz processor. Our feature selection method is tested on the Conference and the WebKB datasets. The proposed ACO based feature selection method is run for 250 iterations, since after 250 iterations we observed that there is no improvement on the classification performance. We have determined the number of ants as 30 experimentally, since we observed that 30 ants give satisfactory results for our study.

In our ACO based feature selection algorithm, each ant chooses a predefined number (i.e., *n*) of features. However, in classical ACO based systems, each ant choses features one by one. The reasons for choosing *n* features are that (i) our classification problem has thousands of features and inclusion or removal of one single feature does not make a considerable effect on the classification performance; (ii) choosing features one by one among the thousands of features increases time complexity of the ACO. In the experiments the number *n* is taken as 10, 100, and 500.

In our ACO based feature selection system, we need a classifier to evaluate the fitness of each set of features selected by each ant. For this purpose, we employed C4.5 classifier since in our previous study [54] we compared classification performance of navie Bayes, kNN (i.e., IBk) and C4.5 (i.e., J48) algorithms of Weka data mining tool, and we observed that C4.5 is the best performer for our datasets.

After determining the best set of features by using our ACO based feature selection algorithm, we use the selected features to classify the web pages in the test datasets. We repeat this process (i.e., ACO based feature selection and then classification of test datasets) 5 times, and the best, worst, and average* F*-measure values of these 5 runs are presented in the following sections.

### 4.5. Experiment 1: Selecting Features Only from URLs of Web Pages

Performance of the proposed method for selecting features from only URL addresses of web pages is considered in this experiment. For this purpose, features are extracted only from the URL addresses of web pages in the training datasets. For all classes, *n* features are selected with our ACO based feature selection algorithm, and then test (unseen) web pages are classified with respect to these selected *n* features. To classify test web pages, J48 classifier is used. This process is repeated 5 times and the best, worst, and average classification performance of these 5 runs in* F*-measure values for the selected *n* features for all datasets is given in Table 4. As Course dataset has less than 500 features extracted from URLs (see Table 3), we only select 100 and 10 features from URLs for Course class.

In Table 4, the best* F*-measure values are written in bold face for all classes. According to the experimental results presented in Table 4, we observed that the WebKB dataset includes meaningful URL addresses so that, for all *n* values, web pages from the Course, Project, Student, and Faculty classes are classified at 100% accuracy by using the ACO selected features from the URLs of the web pages. However, this observation is not true for the Conference dataset, as the best* F*-measure value obtained is 0.883. So, we can say that the Conference dataset has less meaningful URL addresses with respect to the WebKB dataset. There is no considerable difference between varying numbers of features for the WebKB dataset. For the Conference dataset, on the other hand, reducing the number of features also reduces the average* F*-measure values of classification of the test web pages.

### 4.6. Experiment 2: Selecting Features Only from <title> Tags

Performance of the proposed method using only <title> tags of Web pages is considered in this experiment. For this purpose, features are extracted only from the <title> tags of web pages in the training datasets. Similar to the first experiment, each ant selects *n* features from the whole feature set that are obtained from <title> tags, and our ACO algorithm returns the best *n* features. After that web pages in the test dataset are classified with respect to these selected *n* features by using J48 classifier. This process is repeated 5 times and the best, worst, and average classification performance for these 5 runs in* F*-measure value for the selected *n* features for all datasets is given in Table 5. As Course dataset has less than 500 features extracted from <title> tags (see Table 3), we only select 100 and 10 features from <title> tags for Course class.

In Table 5, the best* F*-measure values are written in bold face for all classes. According to the results presented in Table 5, the* F*-measure values of the classification of the test web pages by using the ACO selected *n* features are also high for the WebKB dataset, which implies that the WebKB dataset includes meaningful title declarations. However, the title declarations of the Conference dataset are not meaningful as the WebKB dataset. In this experiment, the reduced number of features affects average* F*-measure values negatively for the Course, Student, and Faculty classes; however, for the Project and Conference classes the average* F*-measure values are improved when the number of features is reduced.

### 4.7. Experiment 3: Selecting Features from Bag of Terms Method

In this experiment, features are selected among terms which are extracted by using bag of terms method. In this method, only the terms regardless of their position or tag are taken as features. Our ACO based feature selection algorithm runs 5 times for each dataset, and we obtain 5 sets of best features. Then by using the selected set of features, we classify the test web pages with J48 classifier. The best, worst, and average classification performance in* F*-measure value of these 5 runs for classifying the test web pages for all datasets is given in Table 6.

In Table 6, the best* F*-measure values are written in bold face for all classes. When we compare Tables 4, 5, and 6, we observed that text contents in web pages are more meaningful for classification than URL addresses and titles for the Conference dataset. However, in the WebKB dataset, URL addresses have better features for classification than page contents and titles of web pages. As in the previous experiments,* F*-measure values change with the number of features and the average classification performance decreases when the number of features is decreased.

### 4.8. Experiment 4: Selecting Features from Tagged Terms Method

In this experiment, features are selected among terms which are extracted by using tagged terms method such that each term in each HTML tag is taken as a different feature. As URLs have very discriminating terms for the WebKB dataset, terms from URLs are not taken in this experiment. Our ACO based feature selection algorithm is run 5 times and we obtain 5 sets of best features for each case. Then by using the selected set of features, we classify the test web pages by using the J48 classifier. The best, worst, and average classification performance in* F*-measure value of these 5 runs for ACO selected *n* features is presented in Table 7.

In Table 7, the best* F*-measure values are written in bold face. According to Table 7, as *n* decreases classification performance increases for the Course, Student, Project, and Faculty classes. However, for the Conference dataset, average classification performance is the best for the medium and high *n* values (i.e., 100 and 500).

When the results of these four experiments are compared, we observed that the WebKB dataset (Course, Project, Student, and Faculty classes) is classified with 100% accuracy (i.e.,* F* measure values are 1.0), and the classification performance of the Conference dataset is also satisfactory (i.e.,* F*-measure values are up to 0.952). For the WebKB dataset, when the number of selected features is small (i.e., *n* = 10), the best feature extraction methods are URL only and tagged terms method. For the Conference dataset, on the other hand, the best feature extraction method is bag of terms and then tagged terms method. This result has occurred because of the fact that the URLs of the WebKB dataset have class specific terms; for the Conference dataset on the other hand, URLs do not have class specific terms. Since tagged terms feature extraction method is successful in general cases, we use this feature extraction method in the rest of the experiments.

### 4.9. Experiment 5: Using Other Classifiers for the Test Phase

C4.5 decision tree classifier, which is named J48 in Weka, is employed in our ACO based feature selection system to compute the fitness of the selected features and therefore to update pheromone values and selection probabilities of features. In the test phase of the previous experiments, we also applied J48 classifier to classify the test (unseen) web pages. In this experiment, our aim is to show whether our ACO based selected features give good classification performance with other well-known classifiers, namely, the kNN and the naïve Bayes. For this purpose, we employ kNN, which is named IBk in Weka, and naïve Bayes classifiers in the test phase. In the ACO-based feature selection phase, we employed J48 classifier. It is also possible to employ kNN or naïve Bayes classifiers in the ACO-based feature selection algorithm; however, according to our previous study [54], J48 has better classification performance with respect to kNN and naïve Bayes for our datasets and that is why we prefer J48 in the feature selection process. Using kNN or naïve Bayes in the ACO based feature selection may be considered for the future work. The best, worst, and average classification performance in* F*-measure values of the 5 runs is presented in Tables 8 and 9.

According to Table 8, as the number of features selected decreases, average classification performance increases for the Project, Student, and Conference datasets when IBk classifier is used in the test phase. For the Faculty dataset, the best classification performance is obtained when 100 features are selected.

For the naïve Bayes classifier, as the number of selected features decreases, the classification performance increases as shown in Table 9. According to Tables 7, 8, and 9 the features selected by our ACO based feature selection algorithm which uses J48 classifier give satisfactory classification performance when these features are used to classify new web pages with IBk and naïve Bayes classifiers. However, when the results presented in Tables 7, 8, and 9 are compared, we can say that using J48 in the test phase yields more satisfactory classification, since we choose features in ACO according to the J48 classifier.

### 4.10. Experiment 6: Distribution of Tags in the Selected Feature Subsets

**Figure 3:**
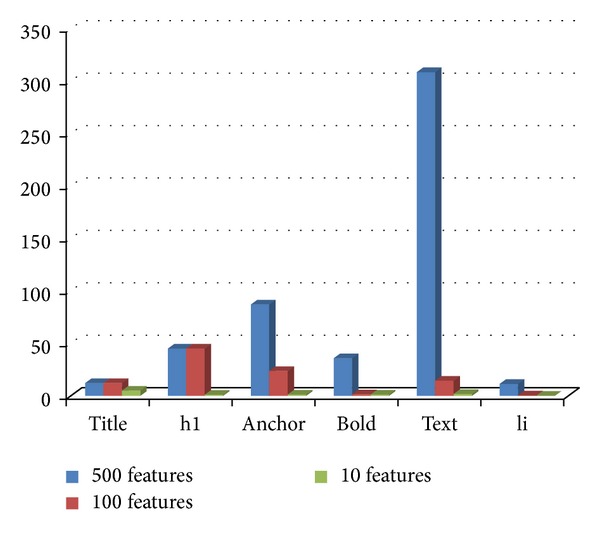
Distribution of the ACO selected tags for course class.

**Figure 4:**
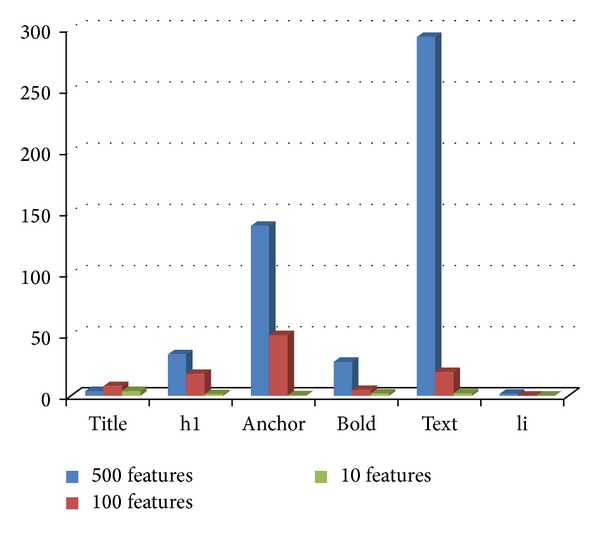
Distribution of the ACO selected tags for project class.

**Figure 5:**
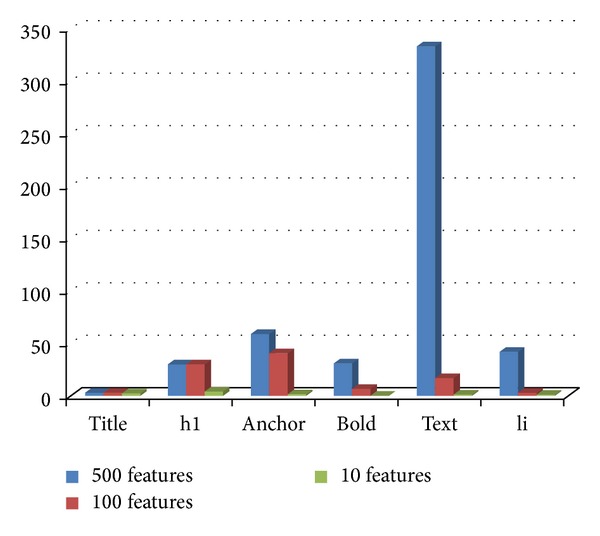
Distribution of the ACO selected tags for faculty class.

**Figure 6:**
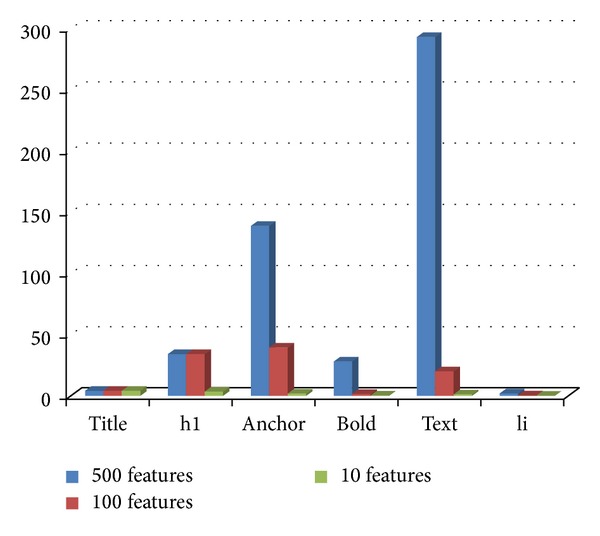
Distribution of the ACO selected tags for student class.

**Figure 7:**
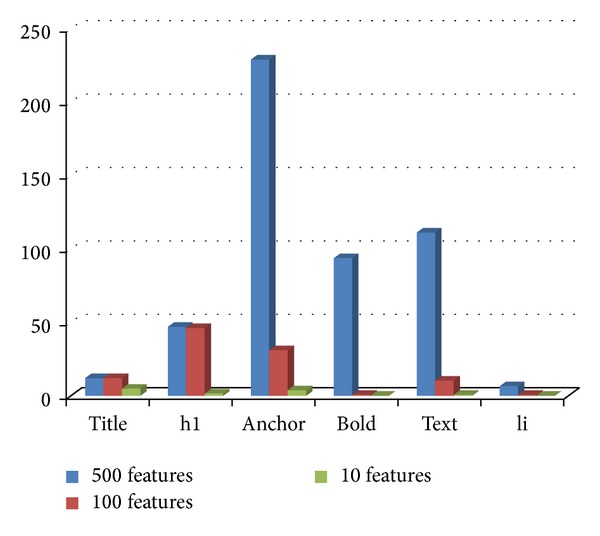
Distribution of the ACO selected tags for conference class.

In this section, we have investigated the distribution of tags in the ACO selected subset of features from tagged terms method for each class. Figures 3, 4, 5, 6, and 7 show tag distributions for the ACO selected features for these five classes. Since URLs are very dominant features for the WebKB dataset as it can be seen from Table 4, we did not include features from URLs in this experiment.

According to Figures 3, 4, 5, 6, and 7, when the number of selected features is small (i.e., 10 to 100) the most discriminative features are obtained from h1, title, and anchor tags. As the number of selected features increases (i.e., 500), features extracted from anchor tag and body text become more dominating. According to our experimental results we observed that using small number of features (10 to 100) is enough to make a good classification for most of our datasets, so instead of using all the features extracted from the web pages, it is enough to use features extracted from URL, h1, title, and anchor tags.

### 4.11. Experiment 7: Effect of ACO Based Feature Selection on Classification Performance in Terms of Accuracy and Running Time

**Figure 8:**
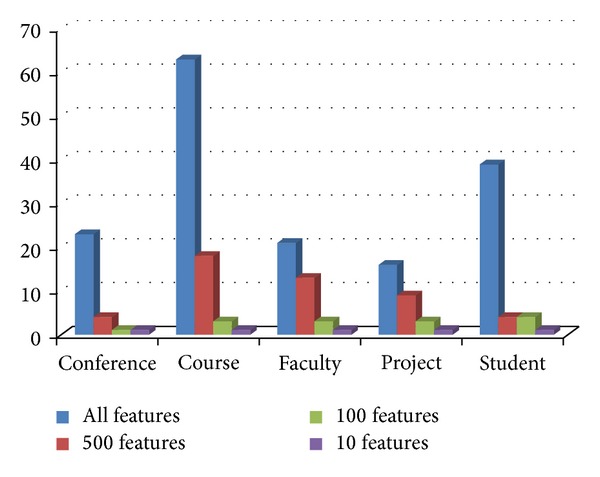
Comparison of time required to classify test pages.

In this section, effect of the proposed ACO based feature selection algorithm on the classification performance has been investigated. For this purpose,* F*-measure values and running time of the classification of the test web pages using the C4.5 classifier with and without the ACO based feature selection are compared. The results of this experiment are presented in Table 10 and Figure 8. In Table 10,* F*-measure values of classification of test web pages with ACO based feature selection and without making any feature selection (i.e., by using all features) are compared for features extracted from tagged terms, URLs, and <title> tags.

When we compared the results presented in Table 10, we can say that classification performance in terms of* F*-measure increases when we used features selected by our ACO based feature selection method from tagged terms. For the features extracted from title tags, classification performance increases with our ACO based feature selection method for the Course, Student, Faculty, and Conference datasets, but* F*-measure value decreases for the Project dataset. For the features extracted from URLs both using all features and making ACO based feature selection give satisfactory classification performance.

In Figure 8, time required to classify test web pages for all classes when C4.5 classifier is used are displayed. As it can be easily seen from the figure, making feature selection reduces the time required to classify new (unseen) web pages sharply without making reduction in classification accuracy (Table 10).

### 4.12. Experiment 8: Comparison of the Proposed Method with the Well-Known Feature Selection Methods

In this experiment, we compared our ACO based feature selection method with two well-known feature selection methods that are information gain and chi square analysis. To accomplish this, we select 10, 100, and 500 features extracted from tagged terms method with information gain (IG) and chi square (Chi) feature selection methods. After that, we classify test datasets by using the selected features. The classification performance in* F*-measure values of our ACO based method, chi Square, and information gain methods is presented in Table 11.

The best* F*-measure values are written in bold face for all cases in Table 11. According to the results given in Table 11, our ACO based feature selection algorithm can select better features with respect to chi square and information gain especially when the number of selected feature is small (i.e., less than 500). As shown in Table 11, when the number of selected features increases, the classification performance of test data decreases, so it is better to use small number of features in terms of classification accuracy and running time (i.e., Figure 8).

### 4.13. Comparison of the Proposed Method with Earlier Studies

Several ACO based feature selection algorithms have been proposed for the UCI dataset. The authors of [22–26, 28, 29, 31, 33, 34] have reported that their proposed ACO based algorithms increase the performance of classification. According to our experimental results, we also observed that ACO based feature selection algorithm improves the classification performance in terms of classification accuracy and time for the WebKB and Conference datasets in general.

In [55], sequential n-grams are used to extract features from the URLs of web pages. The selected features are then classified with maximum entropy and support vector machine separately. The average* F*-measure value is 0.525 for the multiclass classification of the WebKB dataset. Our average* F*-measure value is 1.0 for WebKB dataset when features are selected from URLs and binary classification is made. Based on these results, we can say that our ACO based feature selection with binary class classification has better classification performance for the WebKB dataset.

Özel [56] has used tagged terms as features with a GA based classifier. URL addresses are not used in feature extraction step. Average* F*-measure values are 0.9 and 0.7 for the Course and the Student classes of the WebKB dataset. In our proposed method, on the other hand, average* F*-measure value is increased up to 1.0 for the Course and Student classes. This comparison shows that features from URLs affect the classification performance positively. In addition to this, using ACO based feature selection and then applying the C4.5 classifier perform better than a GA based classifier.

Jiang [57] has proposed a text classification algorithm that combines a k-means clustering scheme with a variation of expectation maximization (EM), and the proposed method can learn from a very small number of labeled samples and a large quantity of unlabeled data. Experimental results show that the average* F*-measure value is 0.7 for WebKB dataset in multiclass classification [57]. This result shows that our ACO-based algorithm with binary classification performs better since it yields higher* F*-measure value.

Joachims [58] has used transductive support vector machines on WebKB dataset with binary class classification and for feature extraction. Bag-of-terms method is used. According to the experimental results of this study, average* F*-measure values are reported as 0.938, 0.537, 0.184, and 0.838 for the Course, Faculty, Project, and Student classes, respectively. Also, these results show that our proposed algorithm has better performance with respect to the SVM algorithm.

Mangai et al. [39] have used Ward's minimum variance measure and information gain for feature selection from the WebKB dataset. 95% accuracy is achieved for Couse dataset with a kNN classifier. However, when we use our ACO-based feature selection algorithm and then apply a kNN classifier to test web pages from the Course dataset, we also obtained 0.951* F*-measure value. When we apply C4.5 classifier after our ACO-based feature selection algorithm, the* F*-measure value increases up to 1.

## 5. Conclusion 

In this study we have developed an ACO-based feature selection system for the web page classification problem. We have used four kinds of feature extraction methods that are, namely, URL only, <title> tag only, bag of terms, and tagged terms. After the feature extraction step, in our ACO based feature selection algorithm, for each ant we select a predefined number of features, and then we evaluate the performance of the selection made by each ant by using the C4.5 classifier. According to the performance of the selected features, we update pheromone values and selection probabilities of the features. This process is repeated until the best features are selected. After selecting the best features, classification of the new (unseen) web pages is made by using the selected feature set. Experimental evaluation shows that using tagged terms as feature extraction method gives good classification performance on the average cases with respect to using bag-of-terms, URL alone, or <title> tag alone methods. Our ACO based feature selection system is able to select better features with respect to well-known information gain and chi square selection methods. The proposed system is effective in reducing the number of features so that it is suitable for classification of high dimensional data. By reducing the feature space, our system also reduces the time required to classify new web pages sharply without loss of accuracy in classification. As future work, performance of our ACO-based feature selection system may be evaluated for the multiclass case.

## Figures and Tables

**Table 1:** Train/test distribution of WebKB dataset for binary class classification.

Class	Train relevant/nonrelevant	Test relevant/nonrelevant
Course	846/2822	86/942
Project	840/2822	26/942
Student	1485/2822	43/942
Faculty	1084/2822	42/942

**Table 2:** Train/test distribution of the conference dataset.

	Train relevant/nonrelevant	Test relevant/nonrelevant
Conference	618/1159	206/386

**Table 3:** Number of features for all classes with respect to feature extraction methods.

Class	Feature extraction method			
	Tagged terms	Bag of terms	〈title〉 tag	URL
Course	33519	16344	305	479
Project	30856	15307	596	686
Student	49452	22245	1987	1557
Faculty	47376	24641	1502	1208
Conference	34952	18572	890	1115

**Table 4:** The best, worst, and average *F*-measure values of classification using ACO selected *n* features from URLs for all classes.

No. of features selected (*n*)	*F*-measure	Dataset				
		Course	Student	Project	Faculty	Conference
10	Best	**1**	**1**	**1**	**1**	0.782
	Avg.	**1**	0.911	0.993	**1**	0.664
	Worst	**1**	0.822	0.986	**1**	0.545

100	Best	**1**	**1**	**1**	**1**	**0.883**
	Avg.	**1**	**1**	**1**	**1**	0.745
	Worst	**1**	**1**	**1**	**1**	0.606

500	Best	—	**1**	**1**	**1**	0.842
	Avg.	—	**1**	**1**	**1**	0.794
	Worst	—	**1**	**1**	**1**	0.745

**Table 5:** The best, worst, and average *F*-measure values of classification using ACO selected *n* features from 〈title〉 tags for all classes.

No. of features selected (*n*)	*F*-measure	Dataset				
		Course	Student	Project	Faculty	Conference
10	Best	**0.983**	**0.92**	**0.891**	**0.947**	0.736
	Avg.	0.966	0.903	0.883	0.901	0.712
	Worst	0.948	0.885	0.875	0.854	0.687

100	Best	**0.983**	**0.92**	0.883	0.939	0.739
	Avg.	0.976	0.916	0.879	0.930	0.713
	Worst	0.980	0.911	0.869	0.921	0.687

500	Best	—	0.92	0.883	0.94	**0.741**
	Avg.	—	0.915	0.879	0.933	0.678
	Worst	—	0.91	0.869	0.922	0.710

**Table 6:** The best, worst, and average *F*-measure values of classification using ACO selected *n* features from bag of terms method for all classes.

No. of features selected (*n*)	*F*-measure	Dataset				
		Course	Student	Project	Faculty	Conference
10	Best	0.926	0.896	0.95	0.862	0.933
	Avg.	0.880	0.780	0.944	0.836	0.926
	Worst	0.834	0.665	0.947	0.811	0.92

100	Best	0.895	0.840	0.934	**0.928**	0.948
	Avg.	0.873	0.821	0.903	0.918	0.940
	Worst	0.851	0.802	0.872	0.908	0.932

500	Best	**0.960**	**0.973**	**0.981**	0.921	**0.952**
	Avg.	0.955	0.969	0.964	0.918	0.949
	Worst	0.950	0.966	0.947	0.915	0.946

**Table 7:** The best, worst, and average *F*-measure values of classification using ACO selected *n* features from tagged terms method for all classes.

No. of features selected (*n*)	*F*-measure	Dataset				
		Course	Student	Project	Faculty	Conference
10	Best	**0.963**	**0.94**	**0.954**	**0.965**	0.921
	Avg.	0.957	0.938	0.95	0.952	0.904
	Worst	0.954	0.937	0.941	0.946	0.877

100	Best	0.902	0.82	0.923	0.928	**0.94**
	Avg.	0.881	0.808	0.887	0.914	0.932
	Worst	0.865	0.79	0.845	0.869	0.911

500	Best	0.9	0.35	0.91	0.356	0.936
	Avg.	0.87	0.345	0.896	0.355	0.932
	Worst	0.885	0.345	0.884	0.354	0.929

**Table 8:** The best, worst, and average *F*-measure values of classification using ACO selected *n* features from tagged terms for all classes when IBk classifier is used in the test phase.

No. of features selected (*n*)	*F*-measure	Dataset				
		Course	Student	Project	Faculty	Conference
10	Best	0.948	**0.929**	**0.937**	0.948	**0.906**
	Avg	0.943	0.914	0.92	0.936	0.888
	Worst	0.929	0.909	0.896	0.915	0.842

100	Best	0.95	0.887	0.918	**0.965**	0.866
	Avg	0.94	0.872	0.906	0.951	0.861
	Worst	0.932	0.858	0.896	0.941	0.855

500	Best	**0.951**	0.902	0.918	0.94	0.844
	Avg	0.944	0.899	0.911	0.938	0.84
	Worst	0.937	0.898	0.908	0.936	0.837

**Table 9:** The best, worst, and average *F*-measure values of classification using ACO selected *n* features from tagged terms for all classes when naïve Bayes classifier is used in the test phase.

No. of features selected (*n*)	*F*-measure	Dataset				
		Course	Student	Project	Faculty	Conference
10	Best	**0.933**	**0.864**	**0.917**	**0.936**	**0.897**
	Avg.	0.856	0.863	0.88	0.926	0.855
	Worst	0.618	0.859	0.809	0.909	0.838

100	Best	0.868	0.38	0.912	0.875	0.797
	Avg.	0.858	0.354	0.901	0.762	0.695
	Worst	0.829	0.324	0.889	0.602	0.645

500	Best	0.543	0.54	0.502	0.527	0.826
	Avg.	0.51	0.539	0.484	0.51	0.821
	Worst	0.478	0.53	0.472	0.501	0.816

**Table 10:** *F*-measure values of the C4.5 classifier with and without making any feature selection.

Dataset	Feature extraction methods					
	Tagged terms		Title		URL	
	ACO selected features	Without feature selection	ACO selected features	Without feature selection	ACO selected features	Without feature selection
Course	**0.963**	0.909	**0.983**	0.898	**1**	1
Faculty	**0.965**	0.911	**0.947**	0.920	**1**	1
Project	**0.954**	0.355	0.891	**0.985**	**1**	1
Student	**0.940**	0.337	**0.920**	0.679	**1**	1
Conference	**0.940**	0.930	**0.741**	0.372	0.883	**0.972**

**Table 11:** Comparison of the proposed method with well-known feature selection methods.

Datasets	No. of selected features								
	10			100			500		
	IG	Chi	ACO	IG	Chi	ACO	IG	Chi	ACO
Faculty	0.89	0.93	**0.96**	0.90	0.91	**0.92**	**0.90**	0.90	0.35
Course	0.91	0.88	**0.96**	0.36	0.36	**0.90**	0.90	**0.91**	0.90
Student	0.80	0.79	**0.94**	0.42	0.42	**0.82**	0.34	0.34	**0.35**
Project	0.83	0.90	**0.95**	0.39	0.39	**0.92**	0.35	0.35	**0.91**
Conference	**0.93**	0.57	0.92	0.94	0.94	**0.94**	0.93	0.93	**0.93**

## References

[1] Qi X, Davison BD (2009). Web page classification: features and algorithms. *ACM Computing Surveys*.

[2] https://maktoob.yahoo.com/?p=us.

[3] Open direct Project http://www.dmoz.org/.

[4] Chen C, Lee H, Tan C (2006). An intelligent web-page classifier with fair feature-subset selection. *Engineering Applications of Artificial Intelligence*.

[5] Shang W, Huang H, Zhu H, Lin Y, Qu Y, Wang Z (2007). A novel feature selection algorithm for text categorization. *Expert Systems with Applications*.

[6] Yang Y, Pedersen JO A comparative study on feature selection in text categorization.

[7] Aghdam MH, Ghasem-Aghaee N, Basiri ME (2009). Text feature selection using ant colony optimization. *Expert Systems with Applications*.

[8] Dorigo M (1992). *Optimization, learning and natural algorithms [Ph. D. thesis]*.

[9] Dorigo M, Maniezzo V, Colorni A (1996). Ant system: optimization by a colony of cooperating agents. *IEEE Transactions on Systems, Man, and Cybernetics B: Cybernetics*.

[10] Liu L, Dai Y, Gao J (2014). Ant colony optimization algorithm for continuous domains based on position distribution model of ant colony foraging. *The Scientific World Journal*.

[11] Mitchell TM (1997). *Machine Learning*.

[12] Chakrabarti S, van den Berg M, Dom B (1999). Focused crawling: a new approach to topic-specific Web resource discovery. *Computer Networks*.

[13] Özel SA, Saraç E Focused crawler for finding professional events based on user interests.

[14] Menczer F, Belew RK Adaptive information agents in distributed textual environments.

[15] Shannon CE (1948). A mathematical theory of communication. *The Bell System Technical Journal*.

[16] Wilbur WJ, Sirotkin K (1992). The automatic identification of stop words. *Journal of Information Science*.

[17] Cover TM, Thomas JA (1991). *Elements of Information Theory*.

[18] Guiasu S (1977). *Information Theory with Applications*.

[19] Yang Y Noise reduction in a statistical approach to text categorization.

[20] Yang Y, Wilbur WJ (1996). Using corpus statistics to remove redundant words in text categorization. *Journal of the American Society for Information Science*.

[21] Reuters Dataset http://kdd.ics.uci.edu/databases/reuters21578/reuters21578.html.

[22] Jensen R, Shen Q (2005). Fuzzy-rough data reduction with ant colony optimization. *Fuzzy Sets and Systems*.

[23] Chen Y, Miao D, Wang R (2010). A rough set approach to feature selection based on ant colony optimization. *Pattern Recognition Letters*.

[24] Huang CL (2009). ACO-based hybrid classification system with feature subset selection and model parameters optimization. *Neurocomputing*.

[25] Sivagaminathan RK, Ramakrishnan S (2007). A hybrid approach for feature subset selection using neural networks and ant colony optimization. *Expert Systems with Applications*.

[26] Vieira SM, Sousa JMC, Runkler TA (2010). Two cooperative ant colonies for feature selection using fuzzy models. *Expert Systems with Applications*.

[27] Nemati S, Basiri ME (2011). Text-independent speaker verification using ant colony optimization-based selected features. *Expert Systems with Applications*.

[28] Kabir MM, Shahjahan M, Murase K (2012). A new hybrid ant colony optimization algorithm for feature selection. *Expert Systems with Applications*.

[29] Akarsu E, Karahoca A (2011). Simultaneous feature selection and ant colony clustering. *Procedia Computer Science*.

[30] Al-Ani A (2007). Ant colony optimization for feature subset selection. *World Academy of Science, Engineering and Technology*.

[31] Wang W, Jiang Y, Chen SW Feature subset selection based on ant colony optimization and support vector machine.

[32] Jain N, Singh JP Modification of ant algorithm for feature selection.

[33] Abd-Alsabour N, Randall M Feature selection for classification using an ant colony system.

[34] Rasmy MH, El-Beltagy M, Saleh M, Mostafa B A hybridized approach for feature selection using ant colony optimization and ant-miner for classification.

[35] Parpinelli RS, Lopes HS, Freitas A (2002). An ant colony algorithm for classification rule discovery. *IEEE Transactions on Evolutionary Computation*.

[36] Holden N, Freitas AA (2004). Web page classification with an ant colony algorithm. *Parallel Problem Solving from Nature—PPSN VIII*.

[37] Jensen R, Shen Q (2006). Web page classification with aco-enhanced fuzzy-rough fetaure selection.

[38] Janaki Meena M, Chandran KR, Karthik A, Vijay Samuel A (2012). An enhanced ACO algorithm to select features for text categorization and its parallelization. *Expert Systems with Applications*.

[39] Mangai JA, Kumar VS, Balamurugan SA (2012). A novel feature selection framework for automatic web page classification. *International Journal of Automation and Computing*.

[40] WEKA http://www.cs.waikato.ac.nz/~ml/weka.

[41] Kim S, Zhang B (2003). Genetic mining of HTML structures for effective web-document retrieval. *Applied Intelligence*.

[42] Ribeiro A, Fresno V, Garcia-Alegre MC, Guinea D (2003). Web page classification: a soft computing approach. *Lecture Notes in Artificial Intelligence*.

[43] Trotman A (2005). Choosing document structure weights. *Information Processing & Management*.

[44] Porter MF (1980). An algorithm for suffix stripping. *Program*.

[45] Dorigo M, Stützle T (2004). *Ant Colony Optimization*.

[46] Bäck T (1996). *Evolutionary Algorithms in Theory and Practice*.

[47] Quinlan JR (1993). *C4.5: Programs for Machine Learning*.

[48] van Rijsbergen CJ (1979). *Information Retrieval*.

[49] Craven M, DiPasquo D, Freitag D Learning to extract symbolic knowledge from the World Wide Web.

[50] CMU http://www.cs.cmu.edu/.

[51] WebKB http://www.cs.cmu.edu/~webkb/.

[52] DBLP http://www.informatik.uni-trier.de/~ley/db/.

[53] Google http://www.google.com.

[54] Saraç E, Özel SA URL tabanlı web sayfası sınıflandırma.

[55] Kan M-Y, Thi HON Fast webpage classification using URL features.

[56] Özel SA (2011). A Web page classification system based on a genetic algorithm using tagged-terms as features. *Expert Systems with Applications*.

[57] Jiang EP Learning to integrate unlabeled data in text classification.

[58] Joachims T Transductive inference for text classification using support vector machines.

